# Effect of Gravity and Task Specific Training of Elbow Extensors on Upper Extremity Function after Stroke

**DOI:** 10.1155/2018/4172454

**Published:** 2018-07-10

**Authors:** Mohamed E. Khallaf

**Affiliations:** Department of Physical Therapy for Neuromuscular Disorders and Its Surgery, Faculty of Physical Therapy, Cairo University, Egypt

## Abstract

**Background:**

In individuals with hemiparetic stroke, reaching with the paretic arm can be impaired by abnormal muscle coactivation. Prior trails for improving upper extremity functions after stroke have underestimated the role of gravitational force in motor planning and execution.

**Objective:**

The aims this trial were to study the effect of gravity as a facilitator for elbow extension and to estimate the immediate and retention effects of task specific training of elbow extensors on upper extremity function after stroke.

**Methods:**

Twenty-six right handed patients with first ever stroke represented the sample of the study. The participants were randomly assigned into two equal groups. The study group received treatment through two phases. Phase one included training for the elbow extensors in an antigravity position. Phase two included a set of task specific exercise for 16 weeks. The control group received traditional passive stretch and range of motion exercises. Manual dexterity and upper limb function were assessed by Nine-Hole Peg Test and Fugl-Meyer upper extremity. Goniometry was used for measuring elbow extension and forearm supination active ranges of motion.

**Results:**

Significant improvements were observed in Nine-Hole Peg Test, Fugl-Meyer upper extremity, and ranges of motion at postintervention and follow-up compared to preintervention at P≤0.05.

**Conclusions:**

The results of this study provide an evidence that antigravity positions can be used as a centrally presented facilitator of elbow extension. Additionally, task specific training was effective in improving upper extremity function and elbow extension range of motion.

## 1. Introduction

Stroke is a universal health problem because of its impact on quality of life and the increase of falls, in addition to many systemic complications [1–3]. Paresis or paralysis of upper extremity (UE) impairs performance of many daily activities such as dressing, bathing, self-care, and writing, thus reducing functional independence. Only 5% of adults regain full arm function after stroke, and 20% regain no functional use [4]. Following stroke, weakness, or paralysis, spasticity and loss of selective individual movement or abnormal coordination were clinically recognized motor deficits. Abnormal coordination can be expressed in the form of abnormal muscle synergies and results in limited and stereotypic movement patterns, which are jerkily, fatiguing, and of limited purpose. Abnormal coupling between elbow flexion with shoulder abduction and elbow extension with shoulder adduction torques were quantitatively characterized and were one of the main factors related to reaching deficits following stroke [5–7].

The ability to extend the elbow is severely degraded with active limb support. This can be related to abnormal neural coupling between shoulder and elbow motoneuron pools [7]. Other studies suggested that this is due to paresis, imbalance of strength between elbow flexors and extensors, or spasticity rather than abnormal neural coupling. As the elbow extended in the horizontal plane, the shoulder abduction torque required to support the arm against gravity increases progressively. Therefore, elbow extension range of motion may be limited as a compensatory mechanism of antigravity shoulder musculature weakness [8, 9].

A few studies with methodological limitations investigated different strategies to reduce the long-term disability and functional impairment from UE paresis. Keller et al. and Ell et al. investigated the effect of transcutaneous triceps stimulation or changes in static proximal joint postures on the abnormal torque patterns in the paretic upper extremity of subjects with hemiparetic stroke [6, 10]. In these studies, the authors only performed isometric or static measurements which did not directly show an increase of the active workspace generated by elbow extensors as it does not explain the effect of mechanical factors such as muscle length changes or muscle strength imbalances across the elbow joint.

Previous studies proved that the gravity is centrally represented in an anticipatory fashion as a driving force during vertical arm movement planning [11–15]. Consequently, the brain can establish relationships between gravity, joint torques, and movements and thus integrate gravitational effect into internal dynamic models [16, 17]. So, antigravity movements can be used as a method for breakdown the neural coupling, increasing muscle strength and coordination of upper extremity movement at the early stages of recovery when the ability of the patient to do movement out of synergy is limited. Once the patient can move independently from synergies, he/she is ready to do motor learning based exercises (task specific training).

Additionally, alternative dynamic strategies are needed to reduce the long-term disability and functional impairment from UE stretch sensitive paresis. Presently, motor learning approaches indicate that therapeutic interventions should be task specific, tailored to the patient's abilities and goals, and provide sufficient active repetition to ensure learning of motor tasks [18]. It can further take advantage of using visual, auditory, and proprioceptive feedback to provide knowledge of results and/or performance [19, 20].

The aims this trial were to study the effect of the gravity as a centrally presented facilitator of elbow extension and to estimate the immediate and retention effects of task specific training of elbow extensor on reaching pattern in patients with stroke. We developed an exercise program that started with stroke patients from the early stages of recovery to the late recovery stages including two phases. In phase one, we used an antigravity position as a method for elbow extensors facilitation, strengthening, and increasing the patients' ability to overcome the abnormal neural coupling. This phase was a preparation for the patient to be able to do the exercises in phase two which require higher level of motor control and coordination without significant interference of weakness, hypertonia, and abnormal neural coupling. Phase two included a set of exercises that were task specific and provided sufficient active repetition with visual, auditory, and proprioceptive feedback to ensure learning of motor tasks. We hypothesized that antigravity positions can be used as a method for facilitation of muscle contraction and if followed by task specific training of elbow extensor may improve UE function in patients with stroke.

## 2. Subjects and Methods

This study included 26 patients who had a diagnosis of first ever stroke in the territory of the middle cerebral artery which was verified by computed tomography or magnetic resonance imaging. Patients were recruited from the department of Physical Therapy for Neuromuscular Disorders and Its Surgery, Faculty of Physical Therapy, Cairo University. All details of study procedures were approved by the ethical committee of Faculty of Physical Therapy, Cairo University. The inclusion criteria were as follow: being able to provide consent form; a duration of illness not less than 3 months; some capacity for active elbow extension with scores of 2 (spasticity present, a facilitator will elicit the limb synergies reflexly) out of 7 on the arm components of the Chedoke McMaster Stroke Assessment (CMSA). Patients with moderate cognitive deficits (assessed by mini mental state examination); visual field defect; visuospatial neglect; finger flexor spasticity (a Modified Ashworth Scale [MAS] score of >2) [21]; spastic dystonia, or any other condition interfering with upper extremity movements other than stroke were excluded from the study. Patients on muscle relaxant were also excluded from the study. For this study, 49 patients with upper extremity deficits after stroke were examined. Thirty patients were eligible but four selected not to participate in the study. Randomization was computer created by a person who was not associated in patient selection. Participants were assigned to two equal groups, study (G_1_, n=13) or control (G_2_, n=13), one week before interventions. Participants were assessed on clinical outcomes at baseline, after intervention (week-16), and at follow-up (week 20). Therapist who did the base line evaluation and follow-up measures had no idea about patients' allocation.

The exercise program of G_1_ participants included two phases depending on the arm stage of motor recovery of the CMSA. Progression criteria from phase one to phase two included the following: spasticity decreases; synergy patterns can be reversed if movement takes place in the weaker synergy first; movements combining antagonistic synergies can be performed when the prime movers were the strong components of the synergy (stage 4 CMSA).

**Figure 1:**
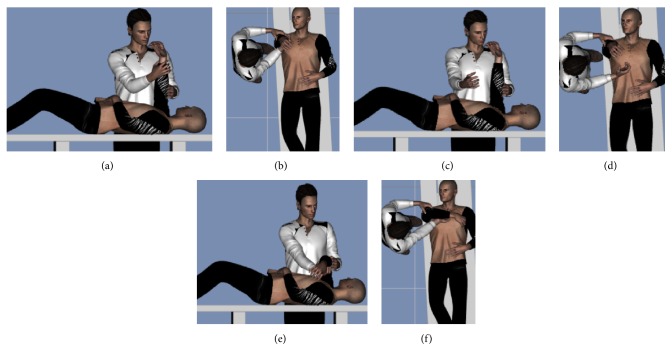
Patient was in supine lying position. (a, b) The affected shoulder was flexed (90°), internally rotated and supported manually by the clinician. The patient turned the head toward the affected side. The affected elbow was passively extended and the forearm was pronated. (c, d) Patient asked to hold the elbow fully extended. (e, f) The elbow was passively flexed and the patients were instructed to actively extend it up with a leading resistance when possible followed by eccentric elbow extension with holding at different ranges ((a, c, e) lateral view and (b, d, f) superior view).

Phase one included use of gravity as a method of facilitation of dynamic elbow extension with isolation of the shoulder movements. Patients were in supine lying position (Figure 1). The affected shoulder was flexed (90°), internally rotated, and supported manually at the arm to maintain this position and to avoid activation of shoulder muscles. The patients were asked to turn the head toward the affected side as a visual feedback and to modulate the flexor's synergy torques through the asymmetrical neck reflex (ATNR) [22]. The affected elbow was passively extended and the radioulnar joints were pronated (Figures 1(a) and 1(b)). Patients were asked to hold the elbow fully extended. At the first 3-5 trials, the patients failed to hold the elbow extended which stimulated the central nervous system to anticipate gravitational loads on the elbow before movement onset and choose trajectories for movements performed against gravity. For the following trials, the patients gradually accomplished the task by holding the elbow extended which opened the door for the elbow extensors to contract concentrically (Figures 1(c) and 1(d)).

Afterward, the elbow was passively flexed and the patients were instructed to actively extend it up against a leading resistance (concentric contraction). This was followed by eccentric elbow extension with holding at different ranges (Figures 1(e) and 1(f)). While the elbow passively extended, patients were asked to lower down the forearm and hand to bend the elbow and hold it for few seconds at different ranges (isometric elbow extensors contraction).

Patients were then asked to repeat the previous steps with the shoulder fully externally rotated, forearm supinated, and the head in neutral position. At this point, all the progression criteria were achieved and the patients were eligible to progress to phase two where task specific exercise can be applied. Phase one might take 9 or 10/16 weeks.

Phase two was for the task specific training (6-7/16 weeks) which included moving the upper extremity between two targets, reaching from lower surface to higher surface, boxing in sitting, picking an object off the floor in sagittal and later frontal plane, and holding ball-shaped weights with different textures or sand-bottle (different weights) with full extension at the elbow in sitting. These activities were done first while the patient was back supported in sitting position and then was without back support throughout five sessions a week; each exercise was repeated 10 times for five sets over 6-7 weeks.

The participants were guided to visualize and copy similar motions by the contralateral arm simultaneously. Analysis of the abnormal pattern of movement with simple explanation was done to understand the differences between normal and abnormal pattern of movements (visual/auditory-motor coactivation). The intended movements were reinforced to be done correctly through clear, simple verbal feedback and encouraged the feel of specific motions as well as applying sensory stimuli simultaneously to movements with care not to overload the patient with excessive or wordy commands especially those with right sided hemiparesis. As initial practice progresses, the patients were asked to self-examine performance and identify problems, specifically, what difficulties exist, what can be done to correct the difficulties, and what movements can be eliminated or refined? Participants in G_2_ received ranges of motion (ROM) exercise, passive stretching, and strengthening of the elbow extensors using elastic bands 5 times a week for 16 weeks.

Three outcome measures were recorded at baseline (1^st^ measurement), postintervention (2^nd^ measurement), and one month after intervention (3^rd^ measurement) for each group. These measures were upper extremity functional recovery using Fugl-Meyer (FM) assessment total upper extremity (FM tUE: A to D motor function), upper extremity (FM UE: A motor function), and dexterity using Nine-Hole Peg Test (NHPT) and elbow extension and forearm supination active ROM. The ROMs were obtained by using universal goniometers [23]. Scores of ROM and NHPT were based on the average of 3 trials of measurement and time taken to complete the test activity.

## 3. Statistical Analysis

Descriptive statistics were calculated to summarize the demographic characteristics of the sample and all outcome measures. Demographic data was compared between groups by *t*-test (*P* < 0.05). Two ways ANOVA (2x3) was used to study interaction effects which represent the combined effects of the applied treatment on the outcome measures. Effects of the applied treatment programs, between groups, were compared using *t*-test with level of significance which was set at *P* <0.05. Repeated measures of ANOVA were employed to calculate, within group, effect of treatment programs in the study or control groups at probability level less than 0.05 (baseline measurements (1^st^ measurement: week_0_), post (2^nd^ measurement: week_16_), and follow-up (3^rd^ measurement: week_20_). In addition, Post hoc pairwise comparisons were used to identify differences between measurements (P<0.05).

## 4. Results

The characteristics of the participants are shown in Table 1. The two groups were matched for age and sex (*p = 0.635, 1.00*), body mass index (*p= 0.581*), and time since onset of stroke (*p = 0.519*). There were nine patients with left sided hemiparesis in both groups (*p = 0.531*). Hypertension and diabetes mellitus were represented in both groups with a percentage of 71% and 68% in group 1 and 67% and 69% in group 2, respectively. Sixty-three percent of the participants in group 1 while 59% of people who were presented in group 2 have cardiac problems.

**Figure 2:**
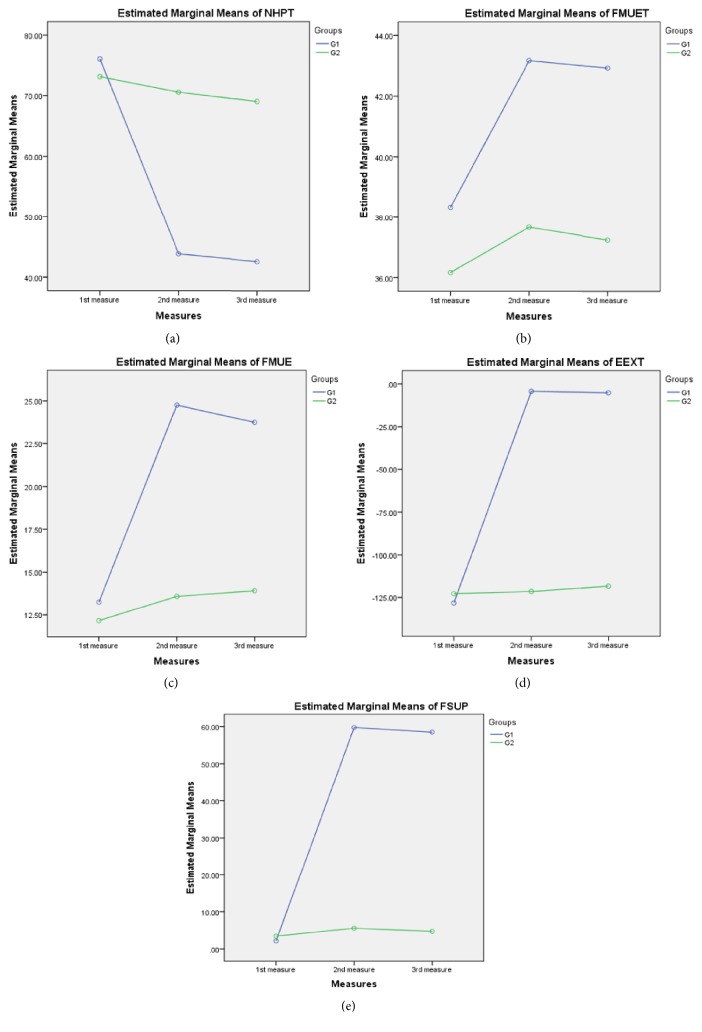
The plot of the mean of (a) Nine-Hole Peg Test (NHPT), (b) total upper extremity Fugl-Meyer tFMUL), (c) FMUL, (d) elbow extension (EEXT), and (e) forearm supination (FSUP) ROMs score for treatment applied on each group and measurements are plotted in a line graph.

A two-way ANOVA (Figure 2) was conducted to examine the effect of applied treatment on each group and different measurements on nine holes pig test. There was a statistically significant interaction between the effects of groups and measurements on NHPT,* F (2, 66) = 14.07*,* P<0.01*. On the same theme, there was a statistically significant interaction between the effects of applied treatment on each group and different measurements on FM tUE,* F (2, 66) = 2.03*,* P<0.01*. For the FM UE (FM A motor function), there was a statistically significant interaction between the effects of applied treatment on each group and different measurements on FM tUE,* F (2, 66) = 40.65*,* P<0.01*. A significant interaction was also found between the effects of applied treatment on each group and different measurements on the measured range of motions: elbow extension (*F (2,66) = 113. 302, P<0.01*) and forearm supination (*F (2,66) = 47. 95, P<0.01*).

Between groups comparison showed that there were nonsignificant differences between the two groups at the baseline measurements of all values including NHPT (*p= 0.582*); FM tUE (FM A to D motor function) (*p= 0.123*); FM UE (FM A motor function) (*p=0.303*); elbow extension (*p=0.067*); and forearm supination (*p= 0.851*). On the other hand, there were statistically significant differences between the second and third follow-up measures (Table 2) of the* NHPT (p= 0.001); FM tUE (p= 0.001); FM UE (p=0.001)*; elbow extension (*p=0.001*); and forearm supination (*p= 0.001*).

A one-way repeated measured analysis of variance (ANOVA) was conducted to evaluate the null hypothesis that there is no change in NHPT scores when measured before, after participation in the study group (*G*_*1*_* n=13*) and one month after interventions. The results of the ANOVA indicated a significant time effect (*Wilks' Lambda=0.16, F (2,10)=42.48, P<0.01)*. Thus, there is an evidence to reject the null hypothesis. On the other hand, it did not show significant time effect on participant of G_2_ (*n=13*) (*Wilks' Lambda=0.88, F (2,10) = 0.698, P>0.520)*. Follow-up comparison in G_1_ indicated that pairwise difference between 2^nd^ and 3^rd^ measurement was not significant,* P>0.05,* suggesting that the effect of the interventions lasts one month after cessation of treatment. Similarly, FM tUE and UE motor function showed significant change in* G*_*1*_ (*Wilks' Lambda=0.46, F (2,10) = 103.35, * and* P=0.001, and Wilks' Lambda=0.21, F (2,10) = 19.06, *and* P=0.001, respectively*) and nonsignificant change in G_2_ (*Wilks' Lambda=0.75, F (2,10) = 1.614,* and* P= 0.247* and* Wilks' Lambda=0.745, F (2,10)= 1.714, P= 0.229,* respectively). In G_1_ post hoc test showed significant differences between the first and second or third measurements* (p<0.05)* but not between the 2^nd^ and 3^rd^ measures* (p=0.463 and 0.132*, respectively).

The results of the one-way repeated-measures ANOVA also showed that, in G_1_, there was a significant effect of interventions on the average elbow extension ROM (*Wilks'Lambda=0.231, F (2,10) = 78.425, *and* p=0.006) *and forearm supination* (Wilks' Lambda=0.013, F (2,10) = 37.066, *and* p=0.005*); however, in G_2_ there, it was a nonsignificant difference (*p=0.098 and 0.148*). Post hoc tests showed that participants of G_1_ showed a significant difference among first and the follow-up measures (*p<0.05) *but it showed a nonsignificant difference between the 2^nd^ and 3^rd^ measures* (p=0.077)*. The nonsignificant difference found between the 2^nd^ and 3^rd^ measurements of the outcome measures indicates that the exercise programs based on the motor learning principles have significant lasting effects.

## 5. Discussion

Statistical analysis of the outcome measures suggests that our hypothesis may have been accurate. The study group demonstrated statistically significant improvements at the immediate and retention levels.

## 6. Gravity as a Facilitator for Elbow Extension

Our results can be attributed to the effect of central presentation of gravity on movement. As the movement performed against gravity, the brain contemplates the direction of movement with respect to gravity and possesses different sensors and mechanisms for sensing torques due to gravity acting on the limb to formulate an appropriate motor plan. This is congruent with the results of Papaxanth et al. who concluded that the CNS considers static forces such as gravity during planning process [14, 15]. This is also consistent with studies reported that gravitational force included into the motor plan and consequently modifies the processes controlling movement programming and execution [14, 15]. It is also in agreement with previous studies which concluded that CNS may regulate the level of activation of the muscle synergies (execution of upper limb movements) according to the perceived arm weight by scaling the amplitude of all the control signals [24, 25].

Our results also can be attributed to the fact that after stroke, muscles acting against gravity have an increased myelination in the contralesional reticulospinal tract [26, 27] and the results indicated that reticulospinal system has the capacity to support bilateral coordination of limb movements using reciprocal actions within a limb or on both sides [28]. So, interventions using antigravity positions and movements may help to increase the neural inputs to the elbow extensors, modulate agonist and antagonist muscle activity in response to a gravitational stimulus, and improve their performance during functional activities.

## 7. Active Range of Motion and Muscle Strength

Hemiparesis is deficiencies in active range of motion (ROM) and in static and dynamic muscle strength. In this study, we presented the results of elbow extension and supination ROM which are directly related to the weakness of the agonist and prime mover muscles [29]. We did not measure the muscle strength as antagonist muscle tone (elbow flexors are the strongest component of the flexor synergy) can contribute to movement deficiencies. Strength imbalances about the elbow joint, specifically weakness of the elbow extensors [30] may contribute to the measurement of abnormal elbow-flexion coupling during maximum shoulder abduction after stroke [31]. Progressive resistive training of elbow extension at phase one improved elbow extension ROM during reaching and functional activities measured by the FM and the NHPT. In this phase, the position of the patient during training allows elbow extension without movement of the trunk (flexion) making the patient attended to the movement without any substitution. This is consistent with previous reports of a positive strengthening effect of progressive strength training in stroke survivors [32].

Furthermore, we used isometric, concentric, and eccentric types of muscle contractions which enable the patient to switch between them as required by the functional and task specific training at phase two of training. Results for our subjects were congruent with the results which reported long-term benefit of progressive resistance training in chronic stroke [33]. Moreover, Harr and Eng showed in a systematic review study that strength training can improve upper limb strength, ROM, and function without increasing tone or pain in individuals with stroke [34].

## 8. Impact of Task Specific Training

In the present study at the second phase of treatment, participants experienced an enhancement in the functional use of the paretic upper extremity because of the task specific, intensive training (TSI) program with sufficient repetitions and structured biofeedback. The results can be attributed to specificity of the intervention, which involved repeated practice of elbow extension while promoting speed and movement accuracy. The results were consistent with studies which reported that task specific training produced statically significant and clinically relevant improvements in the upper extremity motor recovery of the patients with stroke [35] and Sullivan et al. who stated that task specific training in chronic stroke resulted in changes in arm sensation and function that were maintained at 3-month follow-up [36].

Other contributing factors include the establishment of visual/auditory-motor coactivation induced by training. These results are consistent with Villeneuve and colleague who reported that task specific, intensive, and repetitive training intervention can lead to improvements in manual dexterity, finger movement coordination, and functional use of the upper extremity that persist 3 weeks after the intervention [37].

Moreover, the results of this study are constant with Fujii et al., who reported that auditory feedback may facilitate the learning of upper extremity joint coordination pattern when it is provided during practice trials [38]. On the same theme, Kim et al. reported greater improvements in upper extremity functional performance of daily activities and motor control during reaching movements after target reaching training based on visual biofeedback versus traditional rehabilitation [39].

The effects of the treatment persisted at follow-up, indicating that the process of motor relearning has been completed with improved progression, timing, and dexterity of movement. Th can be attributed to the effect of repeated practice of specific motor tasks for the upper limb with visual/auditory-motor coactivation provided during task specific training. This is consistent with Geiger et al. and Van Peppen et al., who stated that motor learning and recovery indicate that intervention should be meaningful, task specific, and tailored to the person's capacity and interests and provide sufficient repetition and challenge to induce training effects [40, 41]. Our results are also in agreement with authors who reported that long-lasting improvements in upper limb function were observed following task specific training [42, 43] who concluded that task specific reach training and environmental enrichment have synergistic effects in rats that persist long after rehabilitation ends.

In this study, we have limitations of a small sample size; one-to-one manual interactions with patients and treatment protocols entail daily therapy for several weeks. Nevertheless, we could show the benefits of using gravity and task specific training. Th trend of changes that we observed warrants further study with a larger sample size, studying the effect of UL partial weight support rehabilitation programs to improve hand function and minimize the effect of gravity on increasing spasticity.

## Figures and Tables

**Table 1:** Subject characteristics, values presented as (mean± SD), and percentages.

	Group 1 *n=13*	Group 2 *n=13*	*P*
Age _(years)_	50.17±2.76	49.5±3.85	0.635

Sex _(males/females)_	8/4	8/4	1.00

BMI_(Kg/m^2^)_	27.74±1.78	27.15±3.01	0.581

Duration of illness _(month)_	21.67±4.68	20.42±5.05	0.519

Paretic left side	5	4	0.531

Hypertension	71%	67%	---

Diabetes	68%	69%	---

Cardiac problems	63%	59%	---

**Table 2:** Between group comparison. Data presented as mean and standard deviations (SD) in seconds (NHPT) and degrees (ROMs).

Variables	Measures	Group 1	Group 2	*t*	*P*
		Mean±SD	Mean±SD		
NHPT (Seconds)	1^st^	75.83±13.02	73.00±13.64	0.568	0.582
	2^nd^	53.42±9.48	70.58±11.39	-4.753	0.001
	3^rd^	55.50±9.94	70.67±11.69	3.371	0.006

FM (tUE)	1^st^	38.33±3.60	36.16±3.66	1.672	0.123
	2^nd^	43.16±2.75	37.66±3.74	4.350	0.001
	3^rd^	42.91±3.28	37.25±3.19	4.507	0.001

FM (UE)	1^st^	13.25±2.38	12.17±2.29	1.080	0.303
	2^nd^	24.08±1.93	13.58±1.68	12.364	0.001
	3^rd^	23.17±2.21	13.92±1.44	11.248	0.001

Elbow Ext. (Degrees)	1^st^	-128.25±5.93	-122.83±6.94	-2.035	0.067
	2^nd^	-4.33±3.06	118.42±6.22	63.066	0.001
	3^rd^	-5.25±2.42	-121.58±4.46	56.388	0.001

Forearm Sup. (Degrees)	1^st^	2.17±14.91	3.83±11.89	-0.192	0.851
	2^nd^	59.75±10.13	5.17±10.21	11.009	0.001
	3^rd^	58.50±9.21	5.50±8.72	12.301	0.001

NHPT: Nine-Holes Peg Test; FM: Fugl-Meyer; tUE: total upper extremity score; UE: upper extremity score; Ext.: extension; Sup.: supination; P is significant at P≤ 0.05.

## Data Availability

The data used to support the findings of this study are available from the corresponding author upon request.
